# Assessment of client satisfaction with pharmacist services at outpatient pharmacy of Tikur Anbessa Specialized Hospital

**DOI:** 10.1371/journal.pone.0224400

**Published:** 2019-10-30

**Authors:** Senait Semegn, Getachew Alemkere

**Affiliations:** Department of Pharmacology and Clinical Pharmacy, School of Pharmacy, College of Health Sciences, Addis Ababa University, Addis Ababa, Ethiopia; College of Pharmacy & Health Sciences, UNITED STATES

## Abstract

**Background:**

Client satisfaction towards the pharmacist services is essential to measure the level of pharmacy services offered to clients and the implementation of pharmaceutical care in the hospital.

**Methods:**

A cross-sectional study was conducted to assess client satisfaction towards the pharmacist service from April 20 to 30, 2019 at OPD pharmacy of Tikur Anbessa Specialized Hospital (TASH). Clients fulfilling the inclusion criteria were interviewed by using a five scale Likert scale. Then data was entered and analyzed using SPSS version 21. The results of the study were presented using table, frequency, and percentage. A binary logistic regression was also employed. The association was declared at p<0.05.

**Result:**

In this study 250 study participants were included. Majority of the participants were males (56.4%, n = 141) with the mean (±standard deviation) age of 38.97±13.73. The mean satisfaction was 51.6%. Study participants perception on pharmacy staff number insufficiency (AOR = 0.32, 95%CI: 0.17, 0.59) and their perception towards the waiting area scored as somewhat fair (AOR = 0.50 (0.27, 0.94) and not convenient (AOR = 0.18 (0.06, 0.56) were negatively associated with their satisfaction.

**Conclusion:**

In this study, study participants have an overall satisfaction of above 50%. Respondent satisfaction for pharmacist approach or communication skill was higher than their satisfaction towards the medication guidance given to them. Study participants perception of the waiting area and staff number sufficiency for the service were significant predictors of their satisfaction. Hence, the TASH administration is expected to improve such pharmaceutical service areas to meet patient demands.

## Introduction

In recent years, the Ethiopian Ministry of Health has made remarkable efforts to improve client satisfaction at the hospital level. The development of the Ethiopian Hospital Reform Implementation Guideline (EHRIG) is one of the basic steps in achieving this goal [1]. Client satisfaction of pharmacist services is an essential tool to measure the level of pharmacy services offered to clients and the implementation of pharmaceutical care in hospitals [2]. Clients who are satisfied with pharmaceutical service are more likely to take medications properly and less likely to change from one health care professional to another [3]. The main way of maintaining client satisfaction is by providing higher quality pharmaceutical service consistently [4].

The obstacles for a better health care service includes poor access to quality medicine, unaffordable cost of drugs, poor education and lack of access for skilled health professionals [2].

In low-income countries, about 75% of hospitals did not have waiting room which caused dissatisfaction with pharmaceutical services. Unavailability of convenient facilities such as chairs and reading materials in the waiting room may compromise client satisfaction. Time taken before they received service is also one factor that affects client satisfaction [5].

Provision of pharmaceutical services is a business making way, therefore, customer satisfaction should be one of the basic goals. While assessing clients' satisfaction with pharmaceutical services it was underlined that the attitude of the pharmacist and provision of information about drugs to clients were important [6].

In Ethiopia, few studies had been conducted on the satisfaction of clients with general health care services. However, there is scarce documentation of opinion of clients satisfaction towards pharmacy services in different parts of Ethiopia [7]. The current study was conducted by the request of the hospital pharmacy department to improve its service to the patient demand level. The current study aims to assess client satisfaction with the pharmacy services, to identify factors affecting client satisfaction and to indicate pharmaceutical service areas for improvement at the Outpatient department (OPD) pharmacy of Tikur Anbessa Specialized Hospital (TASH).

This will help to fill the gap between what the clients need and what they get. The findings are also helpful for identifying specific problems of the service which need improvement in realizing high-quality pharmacy services. Further, it will be a supplement for comparison of practice changes with future studies in TASH OPD pharmacy.

## Methods and materials

### Study setting

The study was conducted at the OPD pharmacy of TASH. TASH is the largest education affiliated referral hospital in the country. It was established in 1972 E.C. The hospital serves approximately 370,000–400,000 clients a year. It has 800 beds, with 169 specialists. According to the management sources, the hospital has 1204 health professionals, of which 74 are pharmacy professionals. Totally 12 pharmacy departments are available which includes pediatric oncology, adult oncology, oncology day care pharmacy, operation room pharmacy, intensive care unit pharmacy, adult emergency pharmacy, pediatric emergency pharmacy, OPD pharmacy, diabetes clinic pharmacy, Anti-retroviral clinic pharmacy, and orthopedic clinic pharmacy. Among these, the OPD pharmacy is the largest outpatient pharmacy with high patient flow.

### Study design and period

A cross-sectional study design was used. The data collection was conducted from April 20 to 30, 2019.

### Inclusion criteria

Clients who were willing to participate in the study, and aged >18 years were included. Clients who were severely sick to respond to the questionnaire and those who were unable to hear or speak were automatically excluded.

### Sample size determination

Initially, the sample size was calculated using a single proportion population formula and determined to be 381. This was based on the assumption of p = 50%. This sample was planned to be addressed with a systematic random sampling method. However, the practical scenario could not allow applying this method. Therefore, the actual sample was collected using a purposive sampling technique. Then, a total of 250 patients were addressed based on the available time and resource. No more than 5 clients refused to take part in the study. The reasons for the refusal were related to the care urgencies. Though none of them discontinued, some of the clients felt reluctant close to the end of the survey.

### Data collection instrument

A structured interview questionnaire was adapted from different related works of literature. The questionnaire includes the client socio-demographic factors, the satisfaction questions and system-related questions that can potentially affect clients’ satisfaction to the pharmacist service (S1 File). The satisfaction questions cover two aspects. The first 6 questions address study participants’ satisfaction towards the pharmacist approach or communication skills. The second 7 questions address participant satisfaction on the medication guide provided by the pharmacists. In all the satisfaction questions, the clients were asked to rate their satisfaction on a five-point scale (1—very satisfied, 2—satisfied, 3—neutral, 4—dissatisfied, 5—very dissatisfied). For descriptive interpretation the five scales were converted into a three scale format by combining very satisfied and satisfied as satisfaction, and dissatisfied and very dissatisfied as dissatisfaction. The questionnaire was prepared in English and then translated to Amharic language and then retranslated to English to ensure consistency. The data was collected through interviewer-administered face to face interview.

### Data quality control and the data collection processes

The questionnaire was pre-tested on 10 clients in the OPD pharmacy before the actual data collection period to check for its appropriateness. Based on the pretest it was corrected and used. No further validation test was performed. Daily review of collected questionnaires for completeness, accuracy, clarity, and consistency of data was carried out. The data was collected by two graduating class clinical pharmacy students on their off-hours and days.

### Data processing and analysis

Data was entered and analyzed using SPSS version 21. The results of the study were presented using descriptive tables. Descriptive statistical methods (frequency, percentages, mean and median) were used to report the data. To identify factors affecting satisfaction, the five-point scale satisfaction score of each study participant was summed and a mean of the sum was calculated. Then, the satisfaction score was dichotomized into satisfaction (less than or equal to 30) and dissatisfaction (above 30). Binary logistic regression was used to identify factors associated with satisfaction. The enter method was applied. The categorical variables were defined using the last variable as a reference. The association was tested using odds ratio and p-value. Those variables with P<0.2 in the univariate analysis were included in the multivariate analysis. The final association was declared using the adjusted odds ratio and p<0.05.

### Operational definition

**Satisfaction:**—a mean satisfaction score of ≤30.

**Dissatisfaction:—**a mean score of > 30.

**Client:**—patient or patient’s caretakers who visit OPD pharmacy during the study period.

### Ethical consideration

Ethical clearance was obtained from the School of Pharmacy Ethical Review Board, Addis Ababa University. Since written consent is not a requirement in such observational studies in our country level, only informed verbal consent was obtained from the clients. The interview was not tape-recorded. Clients were told that participation was voluntary and the right to withdraw from the study at any time was secured. Personal identifiers were not recorded on the questionnaire and all information obtained by face-to-face interview were kept confidential.

## Result

### Socio-demographic characteristics of the clients

In this study, 250 study participants were included. Majority of the participants were males (56.4%, n = 141). Eighty-five (37%) of participants were below the age of 30 years. The mean (± standard deviation) age of the participants was 38.97±13.73. More than half (66.8%, n = 167) of the residents live in urban areas. Most of the participants were married (66.8%, n = 161). Eighty-five (34%) of participants had completed a college degree. More than half (56.8%, n = 142) of the participants purchase the medications for themselves and pay by cash (52.0%). Concerning their previous experiences with the OPD pharmacy, 85.6% (n = 214) participants had more than one visit histories and for 14.4% (n = 36) of them, this was their first visit (**Table 1**).

**Table 1:** Socio-demographic characteristics of study participants.

Variables	Categories	Frequency	Percent
**Sex**	Male	141	56.4
	Female	109	43.6
**Age**	18–30	85	34.0
	31–40	68	27.2
	41–50	42	16.8
	51–60	35	14.0
	> 60	20	8.0
	Mean ± standard deviation	38.97±13.73	
**Residency**	Urban	167	66.8
	Rural	83	33.2
**Marital status**	Single	70	28.0
	Married	161	64.4
	Divorced/Windowed	19	7.6
**Educational level**	Cannot read and write	29	11.6
	Can read and write	17	6.8
	Primary school (grades 1–8)	52	20.8
	Secondary school (grades 9–12)	67	26.8
	Above 12 (college or university)	85	34.0
**Occupation**	Farmer	26	10.4
	Government employee	45	18.0
	Merchant	15	6.0
	Private org. employee	57	22.8
	Student	24	9.6
	Others (Housewife, retirement, not have work)	83	33.2
**Frequency of visit**	First visit	36	14.4
	More than one visit	214	85.6
**Purchase for**	Self	142	56.8
	another person	108	43.2
**Payment status**	Free	119	47.6
	In cash	131	52.4

### Clients perception with pharmacy setting, drug availability, and cost

Medication poor availability (59.6%, n = 149) was the major complaint reported by study participants. Similarly, less than half of the participants agreed on the pharmacy location appropriateness (38.8%, n = 97), the convenience of the counseling area (26.4%, n = 66), the comfort of the waiting area (43.2%, n = 108), and cleanness of the pharmacy (35.6%, n = 89). Among the 250 study participants, 130 get their medication by paying in cash. Among them, 83.8% replied the cost of the medication was affordable. Furthermore, near half of the participants believed that the number of pharmacy staff was not adequate to provide the services (**Table 2**).

**Table 2:** Study participants’ opinion towards the pharmacy setting, medication availability and cost.

Variables (N = 250)	Not	Somewhat/Neutral	Yes
The pharmacy location is convenient	58 (23.2)	95 (38.0)	97 (38.8)
The private counseling area is comfortable and convenient	119 (47.6)	65 (26.0)	66 (26.4)
The waiting area is comfortable and convenient	21 (8.4)	121 (48.4)	108 (43.2)
The dispensary is clean	64 (25.6)	97 (38.8)	89 (35.6)
Medications I need are available	149 (59.6)	6 (2.4)	95 (38.0)
The cost of the medication is fair (n = 130)	19 (14.6)	2 (1.5)	109 (83.8)
The staff numbers are enough to the service	89 (35.6)	15 (6.0)	146 (58.4)

### Satisfaction scores of clients toward pharmacist’s approach

The vast majority of the study participants had a high satisfaction on the politeness (89.6%, n = 224), voice tone (87.2%, n = 218), equitable service delivery (84.4%, n = 211), availability on the workplace (76.8%, n = 192) of the pharmacists. They also had a high satisfaction on the client handling (83.6%, n = 209) (**Table 3**).

**Table 3:** Study participants’ satisfaction towards the pharmacist approach or communication.

Variables	Dissatisfied	Neutral	Satisfied
The politeness and interest of pharmacist was good	23 (9.2)	3 (1.2)	224 (89.6)
Pharmacists provide service equally	34 (13.6)	5 (2)	211 (84.4)
Pharmacists treat the client with dignity and respect	31 (12.4)	10 (4.0)	209 (83.6)
Pharmacy professionals were available during visit	27 (10.8)	3 (1.2)	220 (88.0)
The voice and tone of the pharmacy personnel was clear	29 (11.6)	3 (1.2)	218 (87.2)
Service waiting time in the pharmacy was fair	57 (22.8)	1 (.4)	192 (76.8)

### Clients satisfaction toward pharmacist medication advice

Although the study participants had high satisfaction (76.4%, n = 191) towards the time given, the language of communication (83.2%, n = 208) and the labeling (67.6%, n = 169), they have a lower rate of satisfaction towards most of the guidance they received about their medications. Less than half of the study participants were satisfied with the emphasis given on the medications they received (41.6%, n = 104) and the medication storage conditions (40.0%, n = 100). Furthermore, only about a quarter of the participants were satisfied with the precautions on the medication side effects (26.8%, n = 67) and drug interactions (26.0%, n = 65) (**Table 4**).

**Table 4:** Study participants’ satisfaction towards the pharmacist medication instructions.

Variable	Dissatisfied	Neutral	Satisfied
Counseling time was enough	56 (22.4)	3 (1.2)	191 (76.4)
Give emphasizes on taking medications as told	141 (56.4)	5 (2.0)	104 (41.6)
The pharmacist told me about proper storage of medications	145 (58.0)	5 (2.0)	100 (40.0)
Pharmacist tell about medication precautions and side effects	178 (71.2)	5 (2.0)	67 (26.8)
Medication and drug-drug and drug food interactions	183 (73.2)	2 (0.8)	65 (26.0)
Label readable and understandable instruction	73 (29.2)	8 (3.2)	169 (67.6)
Give administration instructions in understandable language	38 (15.2)	4 (1.6)	208 (83.2)

### The summed satisfaction scores

The mean score of the satisfaction was 30.98 with a standard deviation of 9.11. Using 30 as a cut point, 51.6% of the study participants had a good satisfaction towards the OPD pharmacists service (**Table 5**).

**Table 5:** Study participants' satisfaction score towards pharmacist services.

The summed satisfaction	Frequency (N = 250)	Percent
Mean score (Std. Deviation)	30.98±9.11	
Median score (range)	30.00 (13–60)	
Mode (Median)	29.00 (30.00)	
Satisfaction Score < 30 (Satisfied)	129	51.6%
Satisfaction Score > 30 (Dissatisfied)	121	48.4%

### Factors affecting clients satisfaction

Variables with a p<0.2 in the univariate model as indicated in Table 6 were included in the final multivariate model. Hence, all the socio-demographic variables were excluded from the model. Clients’ response scored as not sufficient on pharmacy staff number sufficiency for the service (AOR = 0.32, 95%CI: 0.17, 0.59) is negatively associated with their satisfaction. Also, clients’ response to the waiting area scored as somewhat fair (AOR = 0.50 (0.27, 0.94) and not convenient (AOR = 0.18 (0.06, 0.56) were negatively associated with the respondent satisfaction (**Table 6**).

**Table 6:** Association test of study participants’ satisfaction towards pharmacist services.

Factors/variables	Frequency (%)		COR	P	AOR (95% C.I.)	P
	Dissatisfied	Satisfied				
Medications I need are available						
Not	82 (67.8)	67 (51.9)	0.52 (0.31, 0.88)	.039	0.65 (0.36, 1.17)	.352
Somewhat/Neutral	2.0 (1.7)	4 (3.1)	1.3 (0.22,7.3)	.015	0.68 (0.09, 4.70)	.150
Yes	37 (30.6)	58 (45.0)	1.00	.785	1.00	.695
**The staffs numbers are enough to the service**						
Not	57 (47.1)	32 (24.8)	0.36 (0.21, 0.62)	.001	0.32 (0.17, 0.59)	.001
Somewhat/Neutral	7 (5.8)	8 (6.2)	0.73 (0.25, 2.12)	.000	0.70 (0.22, 2.22)	.000
Yes	57 (47.1)	89 (69.0)	1.00	.567	1.00	.539
**The pharmacy location is convenient**						
Not	33 (27.3)	25 (19.5)	0.45 (0.23, 0.87)	.018	0.52 (0.24, 1.12)	.163
Somewhat/Neutral	52 (43.0)	43 (33.3)	0.49 (0.27, 0.86)	.017	0.55 (0.27, 1.12)	.096
Yes	36 (29.8)	61 (47.3)	1.00	.015	1.00	.100
**The counseling area is comfortable and convenient**						
Not	52 (43.0)	57 (51.9)	1.1 (0.59, 2.00)	.095	1.53 (0.74, 3.18)	.021
Somewhat/Neutral	39 (32.2)	26 (20.2)	0.56 (0.28, 1.10)	.818	0.57 (0.26, 1.27)	.253
Yes	30 (24.8)	36 (27.9)	1.00	.097	1.00	.169
**The waiting area is comfortable and convenient**						
Not	15 (12.4)	6 (4.7)	0.23 (0.08, 0.63)	.002	0.18 (0.06, 0.56)	.006
Somewhat/Neutral	67 (55.4)	54 (41.9)	0.46 (0.27, 0.78)	.004	0.50 (0.27, 0.94)	.003
Yes	39 (32.2)	69 (53.5)	1.00	.004	1.00	.030
**The dispensary is clean**						
Not	31 (25.6)	33 (25.6)	0.72 (0.38 ,1.38)	.118	1.49 (0.69, 3.24)	.277
Somewhat/Neutral	54 (44.6)	43 (33.3)	0.54 (0.30, 0.97)	.326	0.83 (0.41, 1.65)	.308
Yes	36 (29.8)	53 (41.1)	1.00	.039	1.00	.590
**The cost of the medication is fair**						
Not	10 (8.3)	9 (7.0)	1.1 (0.37, 2.72)	.310	1.36 (0.44, 4.10)	.495
Somewhat/Neutral*	53 (43.8)	69 (53.5)	1.48 (0.88, 2.49)	.963	1.42 (0.79, 2.54)	.593
Yes	58 (47.9)	51 (39.5)	1.00	.139	1.00	.245

*P = significance*, *COR = crude odds ratio*, *AOR = adjusted odds ratio*, *CI = Confidence Interval*, *those respondents taking the medications for free were classified as neutral.

## Discussion

This study was a cross-sectional study designed to assess client satisfaction toward OPD pharmacy service to obtain an insight into the quality of the healthcare service provided at TASH OPD pharmacy. The study was conducted by the request of the hospital pharmacy. Two categories of questions were used to assess the clients’ satisfaction. The first was about the approach or communication of the pharmacists and the second was towards the medication guidance given. The mean satisfaction score was 51.6%. This finding was comparable with Bahirdar Felege Hiwot Referral Hospital study (57.8%) [8]. The current satisfaction level was slightly higher than the Yekatit 12 Referral Hospital study (47%) [9] and slightly lower than the studies in Wolita Sodo teaching hospital (54.2%) [10] and Jimma (57.1%) [11]. In contrast to the current study and the above-cited references in the country, the nationwide survey reported an overall patient satisfaction of 74.5% [2]. The probable reason for such discrepancy may be the parameters considered for satisfaction assessment. The parameters used for assessing patient satisfaction in the nationwide study were dispensing area, dispensing process, personnel skills, the privacy of the setting, and assistance offered to the patient. In this nationwide survey, patient satisfaction towards the TASH dispensary area was below half.

In the current study, although satisfaction reports for the pharmacist approach or communication were consistent and higher, mixed and very low figures were noted towards the medication guidance given to them. This was a warning message that would make the whole medical care effort valueless. Fortunately, the patient satisfaction towards some of the key medication instructions like for the administration instructions (83.2%), the advising time (76.4%) and the label clarity (67.6%) was higher. In support of this finding, the nationwide study on the quality of pharmaceutical service reported a higher knowledge score of patients towards the dose (83.2%), frequency (90.8), and route (94.8%) of administration [2]. In contrast, less than half of the clients in the current study were satisfied on the emphasis given on the medication instructions they received (41.6%, n = 104) and the medication storage conditions (40.0%, n = 100). Furthermore, only about a quarter of the clients in this study were satisfied with the precautions on the medication side effects (26.8%, n = 67) and drug interactions (26.0%, n = 65). Inline to these figures the nationwide study reported as most of the interviewed patients did not know the name, precautions, and storage conditions for the medicines dispensed to them [2]. This implies that the medication guidance was one area that needs to be addressed to improve the overall patient care process. Other studies in Gondar [12] and Addis Ababa also highlighted the need for the improvement in the medication guidance area of the pharmaceutical service.

Study participants satisfaction was also tested for possible determinants. Unlike multiple other studies [9,13,14], all socio-demographic variables were not associated with satisfaction in the current study. On the system-related factors, on the other hand, study participants belief on drug availability, OPD area convenience, and cleanness of the pharmacy were found to be associated with satisfaction in the univariate model. Unfortunately, these factors failed to show a significant association in the multivariate model. In contrast, the study at Yekatit 12 hospital reported a statistically significant association with patient satisfaction and the availability of prescribed drugs in hospital pharmacy (AOR 1.9, 95% CI 1.12–2.88) [9].

Besides, in the current study only study participant belief towards staff number sufficiency and waiting area conformability were significantly associated with the satisfaction score both in the univariate and multivariate models. The significant association on clients’ dissatisfaction towards the waiting area conformability may be explained in line with the nationwide study that included TASH [2]). This study published in 2014 reported a high (more than 50%), patient dissatisfaction towards the dispensary area of TASH. The period between the nationwide study and the current implies the absence of improvements in the dispensary area appropriate to the patient need. Yet, this will remain to be an assignment for the hospital management.

Some of the key limitations of the study were the following. First, the study was conducted in one setting and will not be generalizable to other settings. The small sample size and the non-random sampling method can also compromise the representativeness of the findings. Besides, the use of odds ratio in such a cross-sectional study may overestimate the outcome. Furthermore, the findings of this study might be subjected to social desirability bias because the interview was done on hospital premises.

## Conclusion

In this study, participants have a satisfaction of greater than 50%. Although this figure was comparable to other studies in the country, it was much lower than the nationwide study. Respondents’ satisfaction for pharmacist approach or communication skill was highly encouraging. However, this was compromised by the lower satisfaction report towards the medication guidance given. Finally, participants’ perception of insufficient staff number for the service and waiting area conformability were negatively associated with their satisfaction. The hospital management is recommended to ensure the OPD setting conformability and the delivery of proper and sufficient medication guidance to needy clients. The management will also be expected to employ sufficient staff for proper service delivery.

## Supporting information

S1 File Data collection tool (questionnaire). (DOCX)
