# Supplementary material for: Assessment of client satisfaction with pharmacist services at outpatient pharmacy of Tikur Anbessa Specialized Hospital
Source: PLoS One. 2019 Oct 30;14(10):e0224400. doi: 10.1371/journal.pone.0224400 (PMC6821072; doi:10.1371/journal.pone.0224400)
Supplement: S1 File — (DOCX) [file pone.0224400.s001.docx]

# File 1: Data collection tool (Questionnaire)

# Annex 1

**Verbal consent format**

Hello, my name is . I’m here to undertake a study entitled “assessment of client satisfaction with pharmaceutical services at the Outpatient Pharmacy Department of TASH”. I will request you to listen carefully for the ideas that I will going to read for you and give me your feedback.

The aim of this study is to assess your satisfaction with pharmaceutical Services. You may not get direct benefit for participating in this study but your responses have importance for the identification and correction of major gaps in the pharmacy service. It will take only 10-20 min to participate in this study. Be confident that your name and address will not be recorded. You have also the right to not to answer any question which might be uncomfortable for you. Again I will like to tell you all your answers are confidential and used for research purpose only.

Are you willing to participate in this study?

Yes, I agree

No, I don’t agree

## Annex 2 English version interview questionnaire

**General information**

Serial no.______________

Data collection date________________________________

Signature _______________________________________

1. **Socio-demographic Characteristics of Respondents**

| S.no | Questions | Response |
| --- | --- | --- |
|  | Gender | 1. Male 2. Female |
| 1. 2 | How old are you? | __________years |
|  | Where is your current residence? | 1. Urban 2. Rural 3. Homeless |
| 1. 6 | What is your marital status? | 1. Single 2. Married 3. Divorced/widowed |
| 1. 7 | What is your educational level? | 1. Illiterate 2. Can read and write 3. Primary school (1-8); 4. Secondary school (9-12) 5. Diploma 6. Other specify __________ |
| 1. 8 | What is your main occupation? | 1. Farmer 2. Government employee 3. Merchant 4. Private org. employee 5. Student 6. other __________ |
| 1. 9 | What is your estimated average monthly income | __________birr |
|  | Service sought for during the current visit | 1. For self 2. For others (family/relative/social) |
|  | Payment Status | 1. Free/poverty/insurance covered 2. Paying |
|  | How many times you visited the pharmacist so far? | _________________________ |

1. **Opinion towards the OPD pharmacy setting, medication availability and cost**

| **S.no** | **Questions** | **Not** | **Somewhat/neutral** | **Yes** |
| --- | --- | --- | --- | --- |
|  | The pharmacy location is convenient |  |  |  |
|  | The private counseling area is comfortable and convenient |  |  |  |
|  | The waiting area is comfortable and convenient |  |  |  |
|  | The dispensary is clean |  |  |  |
|  | Medications I need are available |  |  |  |
|  | The cost of the medication is fair |  |  |  |
|  | The staffs numbers are enough to the service |  |  |  |

1. **Satisfaction towards the pharmacist approach or communication**

**1. Strongly Agree, 2.Agree, 3.Neutral/I do not know, 4. Disagree, 5. Strongly Disagree**

| **S.n** | **Question** | **1.** | **2.** | **3.** | **4.** | **5.** |
| --- | --- | --- | --- | --- | --- | --- |
|  | The politeness and interest of pharmacist was good |  |  |  |  |  |
|  | The pharmacist Provide service equally to all patients |  |  |  |  |  |
|  | The pharmacist treat the patient with dignity and respect |  |  |  |  |  |
|  | The pharmacy professionals were available during the time of my visit |  |  |  |  |  |
|  | The voice and tone of the pharmacy personnel was clear |  |  |  |  |  |
|  | The amount of time the pharmacist take to fill my prescription was fair |  |  |  |  |  |

1. **Satisfaction towards the pharmacist medication guidance**

**1. Strongly Agree, 2.Agree, 3.Neutral/I do not know, 4. Disagree, 5. Strongly Disagree**

| **S.n** | **Question** | **1.** | **2.** | **3.** | **4.** | **5.** |
| --- | --- | --- | --- | --- | --- | --- |
|  | The counseling/advising time is sufficient |  |  |  |  |  |
|  | The pharmacist constantly emphasizes on the importance of taking your medications as prescribed |  |  |  |  |  |
|  | The pharmacist told me information about proper storage of medications |  |  |  |  |  |
|  | The pharmacist provide adequate information about medication precautions and side effects |  |  |  |  |  |
|  | The pharmacist provide adequate information about medication drug–drug and drug–food interactions |  |  |  |  |  |
|  | The pharmacist label my medicines in readable and understandable instruction |  |  |  |  |  |
|  | The pharmacist give me medication administration instruction in understandable language |  |  |  |  |  |

**የቃል ስምምነት መጠየቂያ ቅጽ**

ሰላም!፣ ……………….. እባላለሁ፡፡ እዚህ የመጣሁት በዚህ መድሃኒት ቤት ውስጥ የታካሚዎችን እርካታ ምን እንደሚመስል የሚያሳይ ጥናት ለማካሄድ ነው፡፡ የማነብልዎትን ነገሮች በጥንቃቄ አዳምጠዉ እንዲመልሱ በአክብሮት እጠይቃለው፡፡

የዚህ ጥናት ዓላማ በጥቁር አንበሳ ሆስፒታል ውስጥ የሚገኘዉን መድሃኒት ቤት ውስጥ ተጠቃሚ የሆኑ ታካሚዎችን እርካታ ለመገምገም ነው:: በዚህ ጥናት ውስጥ በመሳተፍዎት ቀጥተኛ ጥቅም ላያገኙ ይችላሉ ነገር ግን በዚህ ሆስፒታል ውስጥ ያሉ ዋና ዋና ክፍተቶችን ለመለየት የእርስዎ ተሳትፎ ትልቅ ሚና አለው:: በዚህ ጥናት ለመሳተፍ ከ10-20 ደቂቃ ብቻ ይወስዳል:: ሚስጥርዎትን ለመጠበቅ የእርስዎ ስም እና አድራሻ አይመዘገብም በተጨማሪም የእርስዎ መልስ ምስጢራዊነቱ የተጠበቀ ይሆናል:: እርስዎም የማነብልዎትን ጥያቄ መመለስ ወይም አለመመለስ ይችላሉ :: በድጋሚ ሁሉንም መልሶችዎ ምስጢራዊነታቸዉ እንደሚጠበቅ እና ለጥናት አላማ ብቻ እንደሚውል ልነግርዎት እፈልጋለሁ፡፡

በዚህ ጥናት ለመሳተፍ ፈቃደኛ ነዎት?

አዎ, እስማማለሁ

አይ, አልስማማም

## Annex 3-amharic version interview questioner

**አባሪ 2.አማርኛ ቃለ-መጠይቅ**

አጠቃላይ መረጃ

መለያ ቁጥር ______________

መረጃው የተሰበበት ቀን ________________________ፊርማ ______________________________

1. የምላሽ ሰጪዎች ማህበራዊ-ዳዮሎጂካዊ ባህርያት

| **ተ.ቁ** | **ጥያቄዎች** | **መልሶች** |
| --- | --- | --- |
|  | ፆታ | - - 1. ወንድ     2. ሴት |
|  | ዕድሜዎ ስንት ነው ? | __________ ዓመት |
|  | አሁን መኖሪያዎት የት ነው? | 1. ከተማ 2. ገጠር 3. ቤት አልባ |
|  | የጋብቻ ሁኔታዎ ምንድነው? | 1. ያላገባ 2. ያገባ 3. የተፋታ |
|  | የትምህርት ደረጃዎ ምንድን ነው? | 1. ማንበብና መጻፍ የማይችል 2. ማንበብና መጻፍ የሚችል 3. የመጀመሪያ ደረጃ ትምህርት ቤት (1-8) 4. ሁለተኛ ደረጃ ትምህርት ቤት (9-12) 5. ከ12 በላይ (ኮሌጅ/ዩኒቨርሲቲ) 6. ሌላ ካለ ለይተው ያሳውቁ __________ |
|  | ሥራዎት ምንድነው? | 1. ገበሬ 2. የመንግስት ሠራተኛ 3. ነጋዴ 4. የግል ዘርፍ ሠራተኛ 5. ተማሪ 6. ሌላ ካለ ለይተው ያሳውቁ __________ |
|  | አማካይ ወርሃዊ ገቢዎ ምን ያህል ነው ? | __________ ብር |
|  | መድሃኒቱን እየገዙ ያሉት ለማን ነው? | - - 1. ለራሴ     2. ለሌላ ሰው (ለቤተሰብ/ለማህበራዊ) |
|  | መድሃቱን የሚወስዱት | - - 1. በነጻ (በድህነት መረጃ/በጤና መድህን)     2. በክፍያ |
|  | እስካሁንፋርማሲዉን/የፋርማሲ ባለሙያዉ/ዋን ለመድሃኒት ጉዳይ ለምን ያህል ጊዜ ጎብኝተዋል? | __________ |

1. ምላሽ ሰጪዎች ስለመድኃኒት ቤቱ አቀማመጥ፣ ስመድሃቶች በፋርማሲው መኖርና ስለዋጋቸው ተመጣጣኝነት ያላቸው አስተያየት

| **ተ.** | **ጥያቄዎች** | **1.አይደለም** | **2.በመጠኑ/አላውቅም** | **3. አዎ** |
| --- | --- | --- | --- | --- |
| - 1. 3 | የመድሃኒት ቤቱ አቀማመጥ ለአገልግሎት አመቺ ነው |  |  |  |
|  | የግል ማማከሪያ ክፍሉ ተስማሚና ምቹ ነው |  |  |  |
|  | የፋርማሲው የመቆያ ክፍል ተስማሚና ምቹ ነው |  |  |  |
|  | የፋርማሲ ክፍሉ |  |  |  |
|  | የምፈልጋቸው መድሃኒቶች በፋርማሲው ላይ አሉ/አገኛለሁ |  |  |  |
|  | የመድሃቶች ዋጋ ተመጣጣኝ ነው |  |  |  |
|  | ለታካሚዎች አገልግት የሚሰጡ የፋርማሲ ባሙያዎች ቁጥር ለአገልግሎቱ በቂ ነው፡፡ |  |  |  |

1. ታካሚዎች በፋርማሲ ባሙያዎች አቀራረብ ወይም የተግባቦት ክህሎት ላይ ያላቸው እርካታ

1. በጽኑ እስማማለሁ፣ 2. እስማማለሁ፣ 3. አላውቅም፣ 4. አልስማማም፣ 5.በከፍተኛ ሁኔታ አልስማማም

| **ተ.ቁ** | **ጥያቄዎች** | **1** | **2** | **3** | **4** | **5** |
| --- | --- | --- | --- | --- | --- | --- |
|  | የፋርማሲ ባለሙያ/ዋ ትህትና ጥሩ ነበር |  |  |  |  |  |
|  | የፋርማሲ ባለሙያ/ዋ ታካሚዎቹን በክብር እና በአክብሮት ያስተናግዳል /ታስተናግዳለች |  |  |  |  |  |
|  | የፋርማሲ ባለሙያ/ዋ ለሁሉም ታካሚዎች አገልግሎት በእኩል ይሰጣል/ትሰጠኛለች |  |  |  |  |  |
|  | የፋርማሲ ባለሙያ/ዋ በሄድኩበት ሰአት ተገኝቷል/ታለች |  |  |  |  |  |
|  | የፋርማሲ ባለሙያ/ዋ ድምፅ እና ድምፀ ቅላፄ ግልጽ ነበር |  |  |  |  |  |
|  | ባለሙያው መድሃኒቴን አዘጋጅቶ ለመስጠት (ከባሙያው መድሃኒት ለመውሰድ) የሚፈጅብኝ ጊዜ በቂ ነው |  |  |  |  |  |

1. ታካሚዎች የፋርማሲ ባለሙያው በሚሰጣቸው የመድሃኒት መረጃ ላይ ያላቸው እርካታ

1. በጽኑ እስማማለሁ፣ 2. እስማማለሁ፣ 3. አላውቅም፣ 4. አልስማማም፣ 5.በከፍተኛ ሁኔታ አልስማማም

| **ተ.ቁ** | **ጥያቄዎች** | **1** | **2** | **3** | **4** | **5** |
| --- | --- | --- | --- | --- | --- | --- |
|  | ለማማከር የሚሰጠው ጊዜ በቂ ነው |  |  |  |  |  |
|  | የፋርማሲ ባለሙያው/ዋ መድሃኒቴን በታዘዘልኝ መሰረት መውሰዴ አስፈላጊነት እና ጥቅሙን ደጋግሞ ያሳስበኛል/ታሳስበኛለች |  |  |  |  |  |
|  | የፋርማሲ ባለሙያው/ዋ ስለ መድሀኒቴ ተስማሚ የአቀማመጥ ሁኔታ ይነግረኛል/ትነግኛለች |  |  |  |  |  |
|  | የፋርማሲ ባለሙያዉ/ዋ የመድሃኒቴን ቅድመ ጥንቃቄ እና  ስለ ጎንዮሽ ጉዳቶቹ በቂ መረጃ ይሰጠኛል/ትሰጠኛለች |  |  |  |  |  |
|  | የፋርማሲ ባለሙያው/ዋ መድሃኒቴ ከሌላ መድሃኒት ጋር እና ከምግብ ጋር ስላለው ትስስር በበቂ ሁኔታ ያስረዳኛል/ታስረዳኛለች |  |  |  |  |  |
|  | የፋርማሲ ባለሙያው /ዋ መድሃኒቶቼን በቀላሉ ሊነበብ በሚችል እና በቀላሉ ለመረዳት በሚያስችል ጽሁፍ ይጽፋል/ትጽፋለች |  |  |  |  |  |
|  | የፋርማሲ ባለሙያው /ዋ ስለ መድሀኒቴ አውሳሰድ ግልጽ በሆነ መንገድና ቋንቋ ይነግረኛል/ትነግኛለች |  |  |  |  |  |
